# Study of the Head of Human Dry Radii in a Medical College of Nepal: A Descriptive Cross-sectional Study

**DOI:** 10.31729/jnma.4814

**Published:** 2020-03-31

**Authors:** Muna Kadel, Trilok Pati Thapa

**Affiliations:** 1 Department of Anatomy, Nepalese Army Institute of Health Sciences, Sanobharyang, Kathmandu, Nepal

**Keywords:** *head*, *neck*, *orthopedics*, *prosthesis*, *radius*

## Abstract

**Introduction:**

Radius is a lateral bone of forearm. Its proximal end forms a part of elbow joint and superior radioulnar joint. Knowledge of the shape and size of radial head is essential for construction of radial head prosthesis. The objective of this study is to study the morphology of head of human dry radii.

**Methods:**

A descriptive cross-sectional study was conducted in human dry radii in the dissection hall of Nepalese Army Institute of Health Sciences, Sanobharyang, Kathmandu, Nepal from September to October 2019. Ethical approval was taken. Altogether, 68 dry bones were enrolled in the study by convenience sampling method. Radial head was studied in respect to anteroposterior and transverse diameter, height at medial and distal end and shape. Mean and standard deviations of the parameters were obtained by using Statistical Package for Social Sciences version 20.

**Results:**

Mean height of radial head at medial and lateral end was 0.91 cm and 0.76 cm respectively. Mean anteroposterior and transverse diameter of radial head were 2.09 cm and 2.02 cm respectively. Most common shape of radial head in this study was circular in 40 (59%) radii followed by elliptical in 23 (34%). Mean depth of the superior articular facet of the radial head was 0.19 mm.

**Conclusions:**

The most common shape of radial head is elliptical but it was found to be circular in this study. This study will be useful for orthopedic surgeons in making prosthesis of radial head.

## INTRODUCTION

Radius is a lateral bone of forearm. It has proximal and distal ends and a shaft. Proximal end includes head, neck and tuberosity. Proximal end forms a part of elbow joint and superior radioulanr joint. Humero-radial joint permits flexion and extension movements at elbow joint and superior radioulnar joint permits supination and pronation movements of forearm. That's why proximal end of radius is very important clinically.^1^

The incidence of radial head fractures constitutes about 1.5% to 4 % of all adult fractures and they are responsible for one-third of all elbow fractures.^2,3^ Comminuted radial head fractures with elbow instability can be treated well with a modular radial head prosthesis.^4^ Knowledge of the shape and size of radial head is essential for construction of radial head prosthesis that is anatomically and biomechanically correct.^5^

The aim of the study was to study the morphology of head of human dry radii.

## METHODS

A descriptive cross-sectional study conducted in the dissection hall of Department of Anatomy, Nepalese Army Institute of Health Sciences, Sanobhyrang, Kathmandu, Nepal from September 2019 to October 2019 after obtaining ethical clearance from Institutional Review Committee of Nepalese Army Institute of Health Sciences (Ref No- 245). Study was conducted on Nepalese human dry radii of both sides present in the dissection hall of Nepalese army Institute of Health Sciences. Properly ossified radii without fracture and any structural abnormities were included in the study. Convenience sampling technique was used to collect data. Sample size was calculated using the formula;

n =Z^2^ × SD^2^/ e^2^

 = (1.96)^2^× (0.21)^2^/(0.05)^2^

 = 67.76

 ≈ 68

Where,

n= sample size
Z= 1.96 at 95 % confidence interval
SD= standard deviation (0.19) (Gupta C et al 2015)^6^
e= margin of error, 5%

Following parameters of the head of radius were measured;

➢ Height of radial head in medial (MH) and lateral (LH) sides The medial and lateral height of the radial head was measured as the distance between the radial lip and the head-neck border
➢ Anteroposterior diameter (APD) and transverse diameters (TD) of the radial head
➢ Depth of Superior Articular Facet (D): scale put over the radial head touching most prominent anterior and posterior rim of radial head and depth in centre was measured

All these measurements were taken with the help of digital vernier caliper of accuracy of 0.01mm. Shape of radial head was observed by visual method and classified as circular, elliptical and irregular. All the observations were recorded and tabulated. The data was analyzed with the help of SPSS version 20 software. The descriptive data analysis was done to find mean and standard deviation of different parameters of radial head.

## RESULTS

Among 44 human dry radii, 22 were of right side and 22 were of left side. Mean height of radial head at medial and lateral end were 0.91cm and 0.76cm respectively. Mean anteroposterior and transverse diameter of radial head were 2.09cm and 2.02cm respectively. Mean depth of the superior articular facet of the radial head was 0.19mm with maximum of 0.29mm and minimum of 0.04cm depth. The mean, standard deviation and range of different parameters of radial head is given (Table 1).

**Table 1:** Various parameters of radial head.

Parameters	Minimum (cm)	Maximum (cm)	Mean (cm)	Std. Deviation
MH	0.59	1.19	0.91	0.13
LH	0.52	0.93	0.76	0.09
APD	1.75	2.60	2.09	0.18
TD	1.61	2.42	2.026	0.17
D	0.12	0.29	0.19	0.04

MH: Medial Height, LH: Lateral Height, APD: Anteroposterior Diameter, TD: Transverse Diameter, D: Depth of superior articular facet

**Figure 1:**
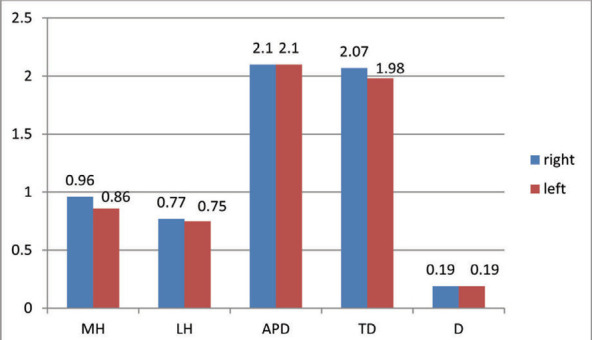
Values of different parameters of radial head in right and left side.

Mean and standard deviations of the different parameters of radial head of right and left side are shown (Figure 1).

**Figure 2:**
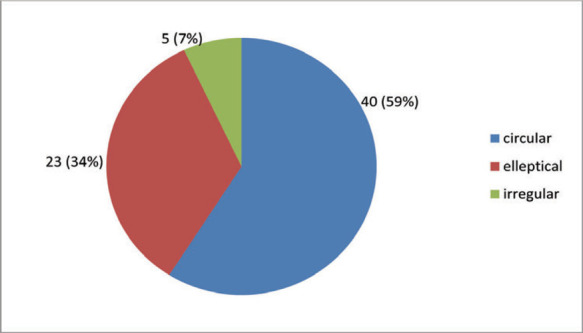
Distrubution of pattern of radical head.

Most common shape of radial head in this study was circular in 40 (59%) radii followed by elliptical in 23 (34%) (Figure 2).

## DISCUSSION

Operative treatment of displaced and comminuted radial head fractures requires internal fixation with plates and screws or replacement with radial head prosthesis when the fracture is unreconstructable. In unstable elbow fractures, accurate implant size is a significant factor to prevent subluxation of the radial head.^6^

In this study, mean anteroposterior and transverse diameter of the radial head was 2.09 cm and 2.02 cm respectively. These findings are supported by the findings of Captier G et al. in which anteroposterior and transverse diameter of the radial head was 2.16 cm and 2.10 cm respectively.7 These values are slightly lesser that is 1.91 cm and 1.85 cm in the study conducted by Gupta C et al.5 Captier et al. also found that the radial head was elliptical in 57% of cases and circular in 43% of cases, but in this study the most common shape was circular in 59% of cases, oval in 34% and irregular in 7% of cases. They also found that biomechanics of the circular shape and elliptical shape are different, involving an adaptation of the angle between the neck and the radial diaphysis. This modification must be taken into concern in the design of radial head prosthesis.^6^

Puchwein et al. also found the mean radial head length on medial and lateral sides as 1.17 and 1.18 cm, respectively, while this study showed the values as 0.91 and 0.76 cm, respectively.7 These values are exact to the values obtained by Gupta C et al.5 These values are less than those reported by Puchwein et al, which may be because they measured the values on CT scan but this study and the study by Gupta C et al. were done on dry bones.5,7 Swieszkowski et al. and Gupta C et al. found the mean depth of articular facet as 0.19 cm, and this study also showed similar value (0.19 cm).^5,8^

This study couldn't be supplemented by imaging techniques. This is the limitation of this study.

## CONCLUSIONS

The most common shape of radial head is elliptical but it was found to be circular in this study. This study will be useful for orthopedic surgeons in making prosthesis of radial head.

## Conflict of Interest:

**None.**
